# Angiosarcoma Developing in an Arteriovenous Fistula after Kidney Transplantation

**DOI:** 10.1155/2017/2426859

**Published:** 2017-08-06

**Authors:** Bruna Natacha Leite Costa, Constantino Fernández Rivera, Maria Calvo Rodríguez, Teresa Hermida Romero, Andrés López Muñiz, Tamara Ferreiro Hermida, Felipe Sacristán Lista, Pilar Iglesias Díaz, Ángel Alonso Hernández

**Affiliations:** ^1^Department of Nephrology, Xerencia de Xestión Integrada A Coruña, A Coruña, Spain; ^2^Department of Pathological Anatomy, Xerencia de Xestión Integrada A Coruña, A Coruña, Spain

## Abstract

After transplantation, the main concerns involve immunosuppression, the prevention and treatment of infections and graft rejection, and tumor prevention. Sometimes the complications that may appear in the arteriovenous fistula are neglected following kidney transplantation. This is the reason why we are presenting the case of an angiosarcoma developing in an arteriovenous fistula after kidney transplantation. It is a very rare case and our goal is to create an alarm so that after kidney transplantation clinicians do not lose sight of the patients' previous history.

## 1. Introduction

Kidney transplantation is considered the best treatment in patients with end stage renal disease [1]. Graft survival has improved in the last years [2] although graft loss after the first year remains constant [3]. The main cause of renal loss is death from tumors, cardiovascular disease, and infections with a functioning graft [4, 5]. Skin, kidney, lung, colorectal, and thyroid cancers are the most reported tumors affecting transplanted patients [6]. Arteriovenous fistula is the best vascular access for hemodialysis when renal failure occurs [7]. After transplantation, the AV fistula should not be closed except in the case of clinical indications such as hand ischemia, recurrent infections, and aneurysmal complications [8]

Here, we report the case of a renal transplant recipient with a high-grade epithelioid angiosarcoma presenting with lung metastasis.

## 2. Case Report

A 70-year-old white woman with a renal transplant performed nine years ago arrived at the emergency room with a dry cough and minimal activity dyspnea. She had no fever, expectoration, or chest pain.

She had been diagnosed with hypertension and dyslipidemia and the origin of the kidney disease was unknown. Before transplantation in 2007, she received hemodialysis for two years due to a right brachiocephalic fistula. In 2012, the brachiocephalic fistula was closed as a consequence of an aneurysm and during the last year she suffered from local pain which was treated with pregabalin and opiates, with no local complications observed. The immunosuppression drugs administered to the patient were tacrolimus, mycophenolate mofetil, and steroids. The renal function remained stable with a serum creatinine level of 1.4 mg/dL (normal range: 0.6–1.2 mg/dL). After transplantation, she suffered from continuous urinary tract infections that were treated with antibiotics, and after a urologic evaluation in 2012, a left kidney nephrectomy was performed because of a pyelonephritis. In March 2015, an anterior colporrhaphy and posterior perineoplasty were also performed to correct a cystocele.

In March 2016, at admission, blood pressure was 159/92 mmHg, temperature was 36°C, and arterial oxygen saturation was 94%. A clinical examination revealed 20 breaths per minute, wheezing and basal hypoventilation, fistula pain, and ankle swelling. The rest of the physical examination was strictly normal with no local findings in the AV-fistula area. Lab tests showed normal C-reactive protein level of 0.77 mg/dL (normal range: 0.0–1.0 mg/dL), serum creatinine of 1.38 mg/dL, and leucocytes count of 7670 (normal range: 4000–11500); PCR herpes viruses 6, 7, and 8 were negative and so were galactomannan antigen, H1N1, and influenza B virus smear. Blood and urine culture were negative as well as cytomegalovirus PCR.

The initial workup showed a diffuse interstitial-alveolar pattern in the chest radiography and hence a piperacillin-tazobactam and a bronchodilator treatment was initiated. The first CT revealed peripheral bilateral infiltrates and minimum pleural effusion. After completing antibiotic therapy, the patient did not improve and a bronchoscopy was performed which showed no atypical cells or signs of infection. BAL and pleural effusion were also tested and were negative for infection and showed no malignancy. A second CT revealed worsening of the infiltrates and pleural thickening which suggested neoplasia. A pulmonary biopsy by videothoracoscopy was performed. It was of an excellent quality and revealed a good representation of the pleura (parietal and visceral), pulmonary parenchyma, vascular areas, and airways.

**Figure 1:**
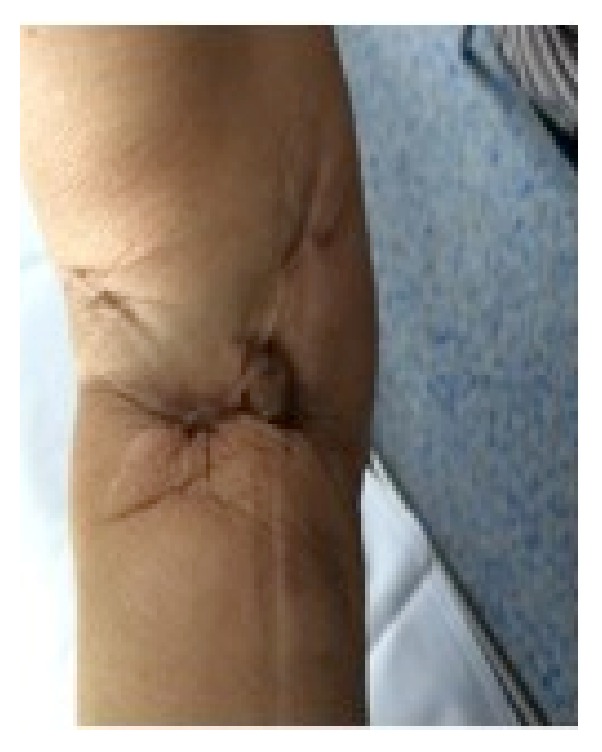
Macroscopic image of the right arm.

**Figure 3:**
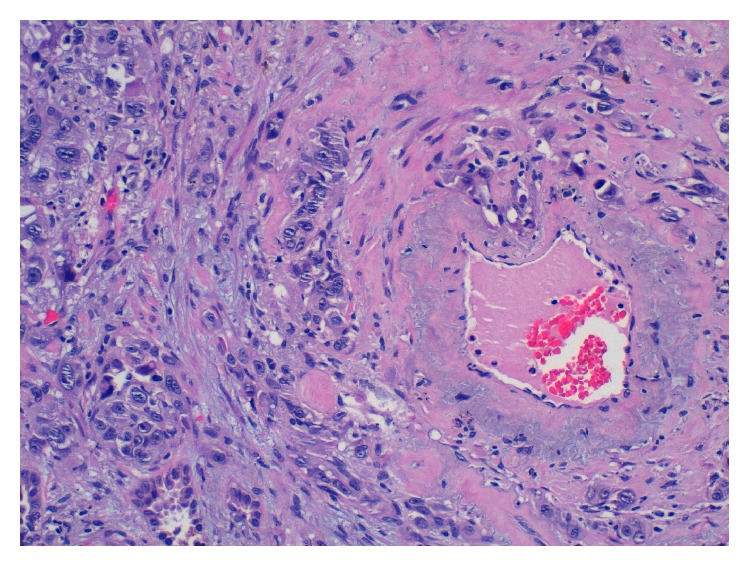
H-E staining of the fistula with large and irregular cells with prominent nucleoli and mitosis.

A first examination of the histologic sections showed a tumor characterized by atypical cells with vascular affectation that invaded the visceral pleura. There were multiple nodular neoformations with hemorrhagic areas showing a circumferential growth that affected blood vessels and bronchioles, which extended to adjacent areas and to the subpleural pulmonary interstitium. Vimentin stain, CD34, CD31, and VIII factor all resulted to be positive. The expression of herpes virus 8 was negative. These results suggested a metastatic mesenchymal tumor. Subsequently, a right arm arteriography revealed a 7 cm mass in the brachiocephalic fistula (Figures 1() and 2()) and a biopsy was performed that was compatible with epitheliod angiosarcoma (Figure 3()). The diagnosis of an arteriovenous fistula angiosarcoma with pulmonary metastasis was carried out.

## 3. Discussion

Tumors, along with cardiovascular disease and infections, are the main causes of death with functioning kidney graft [4, 5]. The incidence of cancer increases after transplantation, but there are some tumors that are more frequent than others. Nonmelanoma skin cancer predominates as the most frequent cancer, followed by lymphoproliferative disorders, especially non-Hodgkin's lymphoma and genitourinary tumors [9]. Colon cancer risk is also higher, and so are several other types of cancer. There has been no demonstrated increased risk for prostate or breast cancer among transplantation recipients compared with the general population [9, 10].

The highest associated risk factors are age, oncogenic virus, and immunosuppressant drugs, most commonly azathioprine, cyclosporin, and steroids [11]. Our patient received tacrolimus, mycophenolate mofetil, and steroids.

Angiosarcomas comprise less than 1% of all sarcomas. They typically arise from endothelial cells in blood or lymph vessels [12]. They are aggressive tumors with median survival of between 11 and 15 months when metastatic disease is presented [13].

Despite angiosarcoma usually having a local affectation of between 20% and 45% present with distant metastatic disease most frequently to the lung [14, 15], there were no local findings in 2012 when the AV fistula was closed.

The prognosis is not related to the chemotherapy response, which suggests that survival depends mostly on histological type, mass size, and presence of metastatic disease [16, 17]. In a recent report of 22 cases of angiosarcoma developing in a fistula, they were all epithelioid sarcomas.

In patients with unresectable metastasis, palliative treatment should be set to prevent symptoms and rapid progression of the disease and to prolong survival. Paclitaxel in monotherapy is ineffective except in the case of angiosarcomas [18]. Our patient survived 6 months after the diagnosis following treatment with paclitaxel.

Although angiosarcoma is rare, it is aggressive and should be suspected when there are clinical changes in the vascular access such as pain, mass, lymphedema, and neurovascular symptoms.

## Figures and Tables

**Figure 2:**
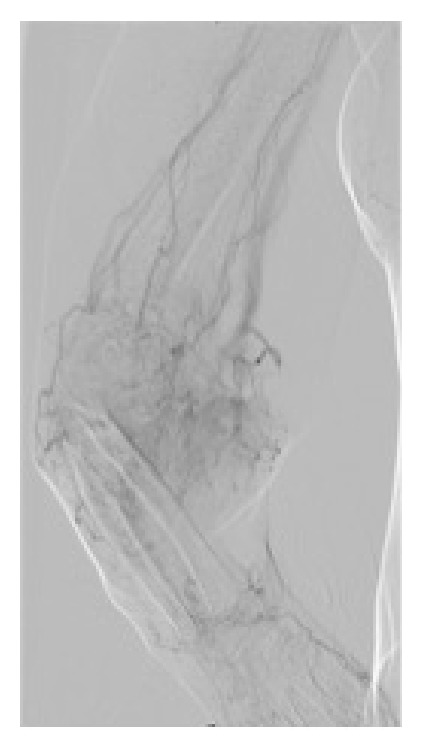
Arteriography of the right arm that revealed a 7 cm mass on the arteriovenous fistula.

## References

[1] Wolfe R. A., Ashby V. B., Milford E. L. (1999). Comparison of mortality in all patients on dialysis, patients on dialysis awaiting transplantation, and recipients of a first cadaveric transplant. *The New England Journal of Medicine*.

[2] OPTN & SRTR Annual data report 2012

[3] Lodhi S. A., Meier-Kriesche H.-U. (2011). Kidney allograft survival: the long and short of it. *Nephrology Dialysis Transplantation*.

[4] Chapman J. R., O'Connell P. J., Nankivell B. J. (2005). Chronic renal allograft dysfunction. *Journal of the American Society of Nephrology*.

[5] El-Zoghby Z. M., Stegall M. D., Lager D. J. (2009). Identifying specific causes of kidney allograft loss. *American Journal of Transplantation*.

[6] Engels E. A., Pfeiffer R. M., Fraumeni J. F. (2011). Spectrum of cancer risk among US solid organ transplant recipients. *The Journal of the American Medical Association*.

[7] Navuluri R., Regalado S. (2009). The KDOQI 2006 vascular access update and fistula first program synopsis. *Seminars in Interventional Radiology*.

[8] Yaffe H. C., Greenstein S. M. (2012). Should functioning AV fistulas be ligated after renal transplantation?. *The journal of vascular access*.

[9] Hall E. C., Pfeiffer R. M., Segev D. L., Engels E. A. (2013). Cumulative incidence of cancer after solid organ transplantation. *Cancer*.

[10] Asch W. S., Bia M. J. (2014). Oncologic issues and kidney transplantation: a review of frequency, mortality, and screening. *Advances in Chronic Kidney Disease*.

[11] Briggs J. D. (2001). Causes of death after renal transplantation. *Nephrology Dialysis Transplantation*.

[12] Qureshi Y. A., Strauss D. C., Thway K., Fisher C., Thomas J. M. (2010). Angiosarcoma developing in a non-functioning arteriovenous fistula post-renal transplant. *Journal of Surgical Oncology*.

[13] van Glabbeke M., van Oosterom A. T., Oosterhuis J. W. (1999). Prognostic factors for the outcome of chemotherapy in advanced soft tissue sarcoma: an analysis of 2,185 patients treated with anthracycline-containing first-line regimens—a European organization for research and treatment of cancer soft tissue and bone sarcoma group study. *Journal of Clinical Oncology*.

[14] Naka N., Ohsawa M., Tomita Y., Kanno H., Uchida A., Aozasa K. (1995). Angiosarcoma in Japan: a review of 99 cases. *Cancer*.

[15] Ramanathan R. C., A'Hern R., Fisher C., Thomas J. M. (1999). Modified staging system for extremity soft tissue sarcomas. *Annals of Surgical Oncology*.

[16] Zagars G. K., Ballo M. T., Pisters P. W. T. (2003). Prognostic factors for patients with localized soft-tissue sarcoma treated with conservation surgery and radiation therapy: an analysis of 1225 patients. *Cancer*.

[17] Skubitz K. M., Haddad P. A. (2005). Paclitaxel and pegylated-liposomal doxorubicin are both active in angiosarcoma. *Cancer*.

[18] Young R. J., Brown N. J., Reed M. W., Hughes D., Woll P. J. (2010). Angiosarcoma. *The Lancet Oncology*.

